# Streptococcus parasanguinis: new pathogen associated with asymptomatic mastitis in sheep.

**DOI:** 10.3201/eid0404.980417

**Published:** 1998

**Authors:** J F Fernández-Garayzábal, E Fernández, A Las Heras, C Pascual, M D Collins, L Domínguez

**Affiliations:** Universidad Complutense, Madrid, Spain. garayzab@eucmax.sim.ucm.es

## Abstract

We describe two unusual cases in sheep of subclinical mastitis caused by Streptococcus parasanguinis. This bacterium has been associated with the development of experimental endocarditis; its presence at relatively high concentrations in apparently healthy sheep milk may pose a health risk in persons with predisposing heart lesions.


### *Streptococcus parasanguinis*: New Pathogen Associated with Asymptomatic Mastitis in Sheep

**J.F. Fernández-Garayzábal,* E. Fernández,* A. Las Heras,* C. Pascual,† M.D. Collins,† and L. Domínguez***
*Universidad Complutense, Madrid, Spain; and †BBSRC Institute of Food Research, Reading, United Kingdom

We describe two unusual cases in sheep of subclinical mastitis caused by *Streptococcus parasanguinis*. This bacterium has been associated with the development of experimental endocarditis; its presence at relatively high concentrations in apparently healthy sheep milk may pose a health risk in persons with predisposing heart lesions.

Ovine mastitis represents a major sanitation and economic problem for both milking sheep farmers and the sheep-milk cheese industry because it reduces milk production and quality. Many microorganisms produce asymptomatic, or subclinical, mastitis in milking sheep. Coagulase-negative staphylococci, particularly *Staphylococcus epidermidis*, are most prevalent (1,2,3), although other bacterial groups have recently emerged as clinically important (4). Streptococci are also responsible for a significant proportion of cases; however, while a large number of species of staphylococci cause subclinical mastitis, the number of streptococci species found to do so is limited, usually to *Streptococcus agalactiae*, *S. uberis,* and *S. dysgalactiae* (3,5).

We report two unusual cases of subclinical mastitis in sheep involving *S. parasanguinis* (6,7). This atypical viridans *Streptococcus* bacterium has been isolated from humans (throat, blood, and urine) (7) but has not been associated with mastitis. The viridans group streptococci are the most common pathogens associated with native valve endocarditis, a disease of increasing medical importance in industrialized countries (8). In patients with this disease, eating and teeth brushing cause low-grade bacteremia, which allows circulating bacteria to adhere to the damaged endocardium (9). Although *S. parasanguinis* has not yet been established as a human pathogen, it has been clearly associated with the development of experimental endocarditis (10). Therefore, the excretion of *S. parasanguinis* at relatively high concentrations in milk from apparently healthy sheep is of concern. Indeed, its presence in certain dairy products, such as nonpasteurized handmade cheeses, may pose a health risk in persons with predisposing heart lesions.

The two isolates in our study were recovered in 1997 from two sheep of an Assaf flock during a bacteriologic survey for determining the prevalence of subclinical mastitis in Madrid (central region of Spain). Affected sheep did not show signs of clinical mastitis or milk abnormalities but had California Mastitis Test (CMT) scores of 2+. CMT estimates the degree of inflammation of the mammary gland by detecting increased numbers of leukocytes in milk (11). The bacteriologic counts of the milk samples were $1 \times 10^4$ CFU/ml in one of the sheep and $1.7 \times 10^3$ CFU/ml in the other. Mammary glands with these characteristics (no clinical abnormalities, apparently normal milk secretion, positive for CMT, and bacteriologically positive) are routinely considered to have subclinical mastitis (1,12). Milk samples were collected and analyzed as described previously (13). After 48 hours of incubation at 37°C under aerobic conditions on Columbia blood agar, pure cultures of α-hemolytic colonies were recovered from both milk samples.

Address for correspondence: J.F. Fernández-Garayzábal, Departamento de Patología Animal I (Sanidad Animal), Universidad Complutense, Facultad de Veterinaria, 28040 Madrid, Spain; fax: 34-91-394-3908; e-mail: garayzab@eucmax.sim.ucm.es.

The isolates are clinically important because both sheep fit the criteria for subclinical mastitis and the isolates were recovered in pure culture.

Both strains were catalase-negative gram-positive cocci, and biochemical identification was attempted with the commercial rapid ID 32 Strep system (Bio-Mérieux S.A.). Both isolates had an identical biochemical profile, which did not correlate with any of the species identified with the ID 32 multisubstrate strip. Sequencing of 16S rRNA genes provided a definitive identification of the streptococci isolates as *S. parasanguinis*. This molecular technique is a powerful method for determining the identity of bacteria that cannot be properly identified by conventional physiologic or biochemical approaches and has proven powerful for describing new bacterial pathogens causing mastitis (13,14). The 16S rRNA gene of each isolate was amplified by polymerase chain reaction (PCR) and was sequenced to determine genotypic identity (13,14). The determined sequences consisted of more than 1,400 nucleotides (representing more than 95% of the total 16S rRNA gene) and were compared with the sequences of other streptococcal species available in the European Molecular Biology Laboratory Database Library. The 16S rRNA gene sequence analysis showed both isolates to be genotypically identical and to display 99.7% sequence similarity with the strain 85-81 of *S. parasanguinis*, which on the basis of DNA-DNA hybridization studies was shown to be a true member of this species (7).

The two *S. parasanguinis* isolates represented 7% of our total streptococci isolated from cases of subclinical mastitis. According to these data, only sporadic cases of mammary gland infections due to *S. parasanguinis* would be expected. However, because many streptococcal isolates are usually identified only to the genus level (2,3,11,15), the diversity of species involved and the incidence of infection due to particular streptococcal species are difficult to know. The same difficulty is true with corynebacteria (4).

The recognition of *S. parasanguinis* as a new animal pathogen causing subclinical mastitis in sheep and the recent recognition of new *Corynebacterium* species associated with this disease (13,14) suggest the need for epidemiologic surveys to determine the range of species diversity, other than coagulase-negative staphylococci, involved in subclinical sheep mastitis. According to recent results, this range appears higher than previously considered. Particular attention should be paid to species potentially pathogenic for humans.

### Acknowledgment

We thank F. Vilas, Director General de Salud Pública de la Comunidad de Madrid, for his encouragement.

Dr. Las Heras is recipient of a long-term predoctoral grant from the Ministerio de Educación y Cultura de España. This work has been partially supported by the Cooperativa Castellana de Ganaderos (Campo Real, Madrid) and by a project from the Comunidad Autónoma de Madrid (Consejería de Educación y Cultura, No. 06G/026/96).

### References

1. Stefanakis A, Boscos C, Alexopoulos C, Samartzi F. Frequency of subclinical mastitis and observations on somatic cell counts in ewes' milk in northern Greece. Animal Science 1995;61:69-76
2. Watkins GH, Burriel AR, Jones JET. A field investigation of subclinical mastitis in sheep in southern England. British Veterinary Journal 1991;147:413-20.
3. Watson DL, Franklin NA, Davies HI, Kettlewell P, Frost AJ. Survey of intramammary infections in ewes on the New England Tableland of New South Wales. Aust Vet J 1990;67:6-8.
4. Fernández-Garayzábal JF, Dominguez L. Corinebacterias como agentes responsables de mastitis subclínicas. In: Abstracts of the XVI Congreso de la Sociedad Española de Microbiología; 1997 Jul 14-17; Barcelona, España. Abstract No. P5.
5. Bor A, Winkler M, Gootwine E. Nonclinical intramammary infection in lactating ewes and its association with clinical mastitis. British Veterinary Journal 1989;145:178-84.
6. Truper HG, Clari L. Taxonomic note: necessary correction of specific epithets formed as substantives (nouns) "in apposition". Int J Syst Bacteriol 1997;47:908-9.
7. Whiley RA, Fraser HY, Douglas CWI, Hardie JM, Williams AM, Collins MD. *Streptococcus parasanguinis* sp. nov., an atypical viridans *Streptococcus* from human clinical specimens. FEMS Microbiol Lett 1990;68:115-22.
8. Scheld W, Sande M. Endocarditis and intravascular infections. In: Mandell G, Bennett J, Dolin R, editors. Principles and practice of infectious diseases. 4th ed. New York: Churchill Livingstone, Inc.; 1995. p. 740-83.
9. Kaye D, Abrutyn E. Prevention of bacterial endocarditis. Ann Intern Med 1991:114:803-4.
10. Burnette-Curley D, Wells V, Viscount H, Munro CL, Fenno JC, Fives-Taylor P, et al. FimA, a major virulence factor associated with *Streptococcus parasanguinis* endocarditis. Infect Immun 1995;63:4669-74.
11. Fthenakis GC. California mastitis test and Whiteside test in diagnosis of subclinical mastitis of dairy ewes. Small Ruminant Research 1995;16:271-6.
12. González-Rodríguez MC, Gonzalo C, San Primitivo F, Cármenes P. Relationship between somatic cell count and intramammary infection of the half udder in dairy ewes. J Dairy Sci 1995;78:2753-9.

13. Fernández-Garayzábal JF, Collins MD, Hutson RA, Fernández E, Monasterio R, Marco J, et al. *Corynebacterium mastitidis* sp. nov., isolated from milk of sheep with subclinical mastitis. Int J Syst Bacteriol 1997;47:1082-5.
14. Fernández-Garayzábal JF, Collins MD, Hutson RA, González I, Fernández E, Domínguez L. *Corynebacterium camporealensis* sp. nov., associated with subclinical mastitis in sheep. Int J Syst Bacteriol 1998;48:463-8.
15. Cruz M, Serrano E, Montoro V, Marco J, Romero M, Baselga, R, et al. Etiology and prevalence on subclinical mastitis in the Manchega sheep at mid-late lactation. Small Ruminant Research 1994;14:175-80.
